# Calcitonin gene-related peptide pre-administration acts as a novel antidepressant in stressed mice

**DOI:** 10.1038/srep12559

**Published:** 2015-08-07

**Authors:** Narumi Hashikawa-Hobara, Takumi Ogawa, Yusuke Sakamoto, Yumi Matsuo, Mami Ogawa, Yoshito Zamami, Naoya Hashikawa

**Affiliations:** 1 Department of Life Science, Okayama University of Science, 1-1 Ridai-cho, Kita-ku, Okayama 700-0005, Japan; 2 Medicinal Drug Design, Graduate School of Medicine, Dentistry and Pharmaceutical Sciences, Okayama University, 1-1-1 Tsushima-naka, Kita-ku, Okayama 700-8530, Japan

## Abstract

Calcitonin gene-related peptide (CGRP) is a neuropeptide that has potent vasodilator properties and is involved in various behavioral disorders. The relationship between CGRP and depression-like behavior is unclear. In this study, we used chronically stressed mice to investigate whether CGRP is involved in depression-like behavior. Each mouse was exposed to restraint and water immersion stress for 15 days. After stress exposure, mice were assessed using behavioral tests: open field test, forced swim test and sucrose preference test. Serum corticosterone levels, hippocampal proliferation and mRNA expression of neurotrophins were measured. After stress exposure, mice exhibited depression-like behavior and decreased CGRP mRNA levels in the hippocampus. Although intracerebroventricular CGRP administration (0.5 nmol) did not alter depression-like behavior after 15-day stress exposure, a single CGRP administration into the brain, before the beginning of the 15-day stress exposure, normalized the behavioral dysfunctions and increased nerve growth factor (*Ngf*) mRNA levels in stressed mice. Furthermore, in the mouse E14 hippocampal cell line, CGRP treatment induced increased expression of *Ngf* mRNA. The NGF receptor inhibitor K252a inhibited CGRP’s antidepressant-like effects in stressed mice. These results suggest that CGRP expression in the mouse hippocampus is associated with depression-like behavior and changes in *Ngf* mRNA levels.

Depression is a major psychiatric disorder that is associated with high rates of suicide, and is considered one of the most important causes of human disability. Excessive exposure to stressful life events induces the onset of behavioral disorders, including depression and post-traumatic stress disorder. The mechanisms underlying the psychopathology of depression are multifaceted, however, it is known to be accompanied by a decrease or impairment of neurogenesis in the hippocampus^1^,^2^. Therefore, mechanistic investigations of depression that target neurogenesis in the hippocampus are crucial. Calcitonin gene-related peptide (CGRP), a potent vasodilator^3^ and neurotransmitter in the central nervous system^4^, is a 37-residue amino acid. CGRP receptors are distributed in the hypothalamus, central gray matter, ventromedial nucleus of the thalamus, amygdala, hippocampus and dentate gyrus^5^. CGRP-containing neurons are found in the hypothalamus, preoptic area, amygdala, thalamus, hippocampus (CA3 pyramidal cells), and dentate gyrus granule cells^3^,^6^. CGRP is reported to be involved in various behaviors suggestive of anxiety. Intracerebroventricular (i.c.v.) CGRP infusions evoke fear-like freezing^7^ and anxiety behavior^8^, and improve learning and memory processing^9^. However, it is less well understood whether CGRP is involved in depression-like behavior. Clinical research shows that CGRP levels may be altered in depressed patients^10^. With this in mind, the aim of the present study was to examine whether CGRP is involved in behavioral development using a 15-day stress exposure model in mice.

The role of neurotrophins, including brain-derived neurotrophic factor (BDNF), nerve growth factor (NGF), neurotrophin-3 (NT-3), neurotrophin-5 (NT-5), ciliary neurotrophic factor, (CNTF) and glial cell line-derived neurotrophic factor (GDNF) in depressive-like behavior have been well studied^11^,^12^,^13^,^14^,^15^,^16^. Their presence is required in the adult central nervous system for maintenance of neuronal functions and neurogenesis^17^,^18^. Administration of neurotrophins normalizes depression-like behavior and increases brain monoamine neurotransmitter levels in chronic mild stress models in rats^15^,^18^. In the present study, we determined the molecular mechanisms by which CGRP changes affect depression-like behavior in mice, particularly focusing on NGF.

## Results

### 15-day stress exposure induced depression-like behavior with reduced hippocampal cell proliferation and decreased *Cgrp* mRNA expression in mice

**Figure 1:**
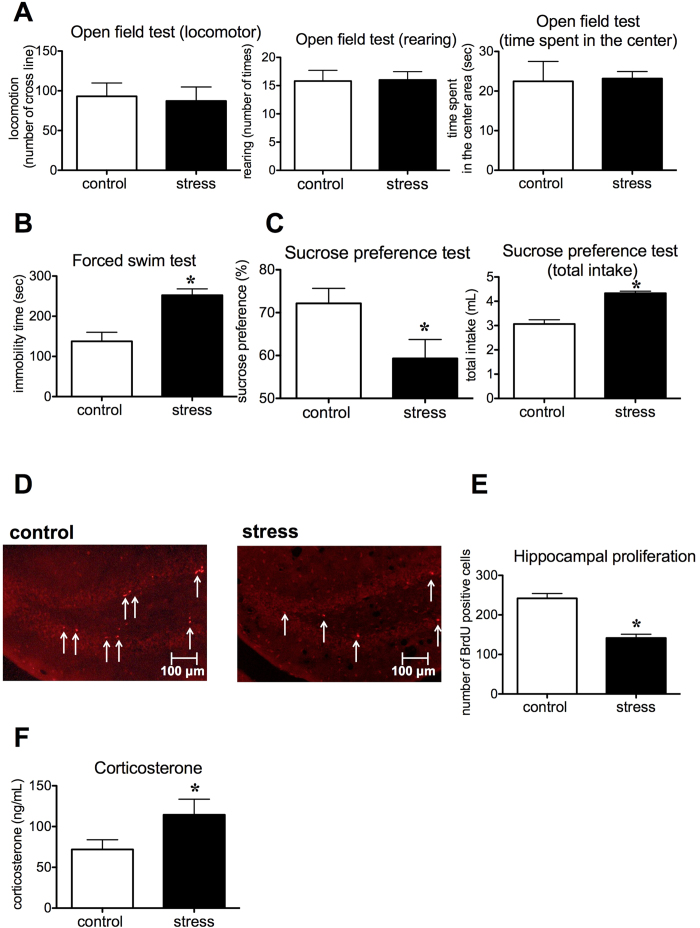
Stressed mice display depression-like behavior and changes in hippocampal proliferation. (**A**) Open field test analysis showing locomotor activity, rearing activity and time spent in the center area following 15-day stress exposure in mice (control n = 5, stress n = 5). (**B**) Immobility times in the forced swim test (control n = 5, stress n = 5). (**C**) Sucrose preferences and total fluid intake in the sucrose preference test (control n = 7, stress n = 7). (**D**) Photomicrographs of the dentate gyrus showing representative BrdU-positive cells. Scale bar indicates 100 μm. Arrows show immunopositive cells. (**E**) The number of BrdU-positive cells (control n = 4, stress n = 3). (**F**) Serum corticosterone level (control n = 7, stress n = 7). Each bar indicates the mean ± S.E.M. *P < 0.05 vs. control.

In the open field test, locomotor activity, rearing activity and time spent in the center area by stressed and non-stressed mice were not significantly different (P = 0.409, P = 0.468, P = 0.4484 respectively, Fig. 1A). Immobility times in the forced swim test in stressed mice were significantly longer than non-stressed mice (P = 0.0016, Fig. 1B). In the sucrose preference test, which measures anhedonic-like deficits, sucrose preferences were significantly lower in stressed than non-stressed mice (P = 0.0207, Fig. 1C), with significant increases in total fluid consumption (P = 0.0001, Fig. 1C). To examine the effects of 15-day stress on hippocampal cell proliferation, we performed BrdU immunohistochemistry. Stressed mice showed fewer BrdU-positive cells in the subgranular zone of the dentate gyrus compared with non-stressed control mice (Fig. 1D). Additionally, the number of BrdU-positive cells in the granule cell layer of the dentate gyrus was significantly lower in stressed mice than in non-stressed control mice (P = 0.0009, Fig. 1E). Serum corticosterone levels, which are induced by stress exposure^19^, were significantly increased in stressed mice compared with non-stressed mice (P = 0.042, Fig. 1F). These results suggest that 15-day stress exposure induced depression-like behavior with reduced hippocampal cell proliferation and increased corticosterone levels.

**Figure 2:**
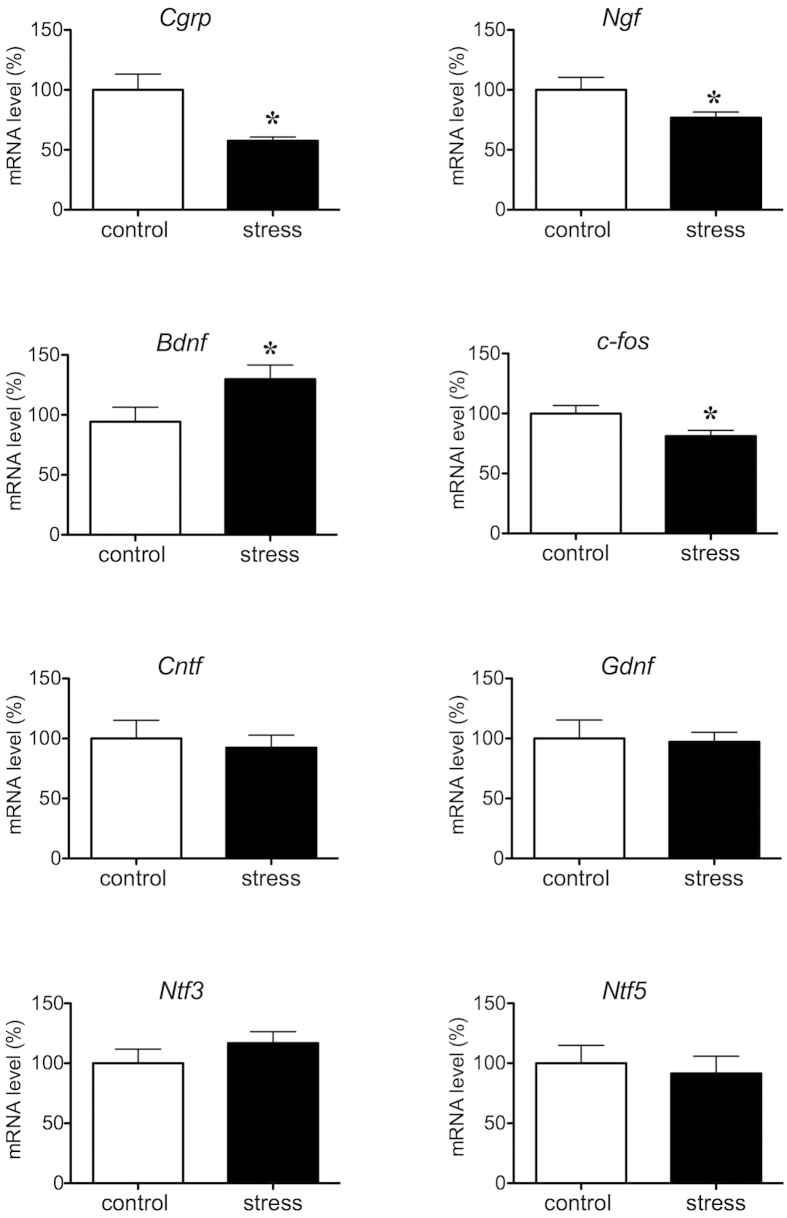
Effects of 15-day stress exposure on mRNA level of *Cgrp*, *c-fos* or neurotrophic factors in the mouse hippocampus. Quantitative real-time RT-PCR analysis showing the expression levels of *Cgrp, Ngf, Bdnf, c-fos, Cntf, Gdnf, Ntf3* and *Ntf5* (control n = 8, stress n = 7). Each bar indicates the mean ± S.E.M. *P < 0.05 vs. control.

To determine whether CGRP was responsible for depression-like behavior, quantitative RT-PCR was used to detect mRNA changes after stress in the hippocampus. *Cgrp* mRNA was significantly decreased by approximately two-fold when compared with non-stressed mice (P = 0.0059, Fig. 2). mRNA levels of *Ngf*, which encodes a neurotrophic factor, were also significantly lower in stressed than non-stressed mice (P = 0.0279), whereas *Cntf* (P = 0.3428), *Gdnf* (P = 0.4389), *Ntf3* (P = 0.1496) and *Ntf5* (P = 0.3746) mRNA levels were not significantly different. Interestingly, only *Bdnf* was significantly increased by stress exposure (P = 0.0308, Fig. 2). Furthermore, 15-day stress exposure induced significant decreases in neuronal activation, as determined by *c-fos* mRNA levels (P = 0.0258, Fig. 2).

### CGRP pre-administration rescued depression-like behavior through *Ngf* mRNA expression

**Figure 3:**
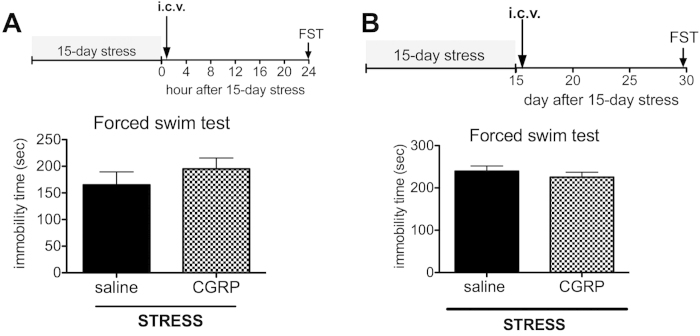
Effects of CGRP i.c.v. administration in mouse depression-like behavior in the forced swim test. Mice were administered saline or CGRP (0.5 nmol). (**A**) On the day following the 15-day stress exposure (day 16), mice were administered saline or CGRP and 24 h later, assessed in the forced swim test (saline n = 5, CGRP n = 6). (**B**) Mice were administered saline or CGRP (0.5 nmol) i.c.v. on the day following the 15-day stress exposure (day 16) and the forced swim test was assessed on day 30 (saline n = 6, CGRP n = 7).

**Figure 4:**
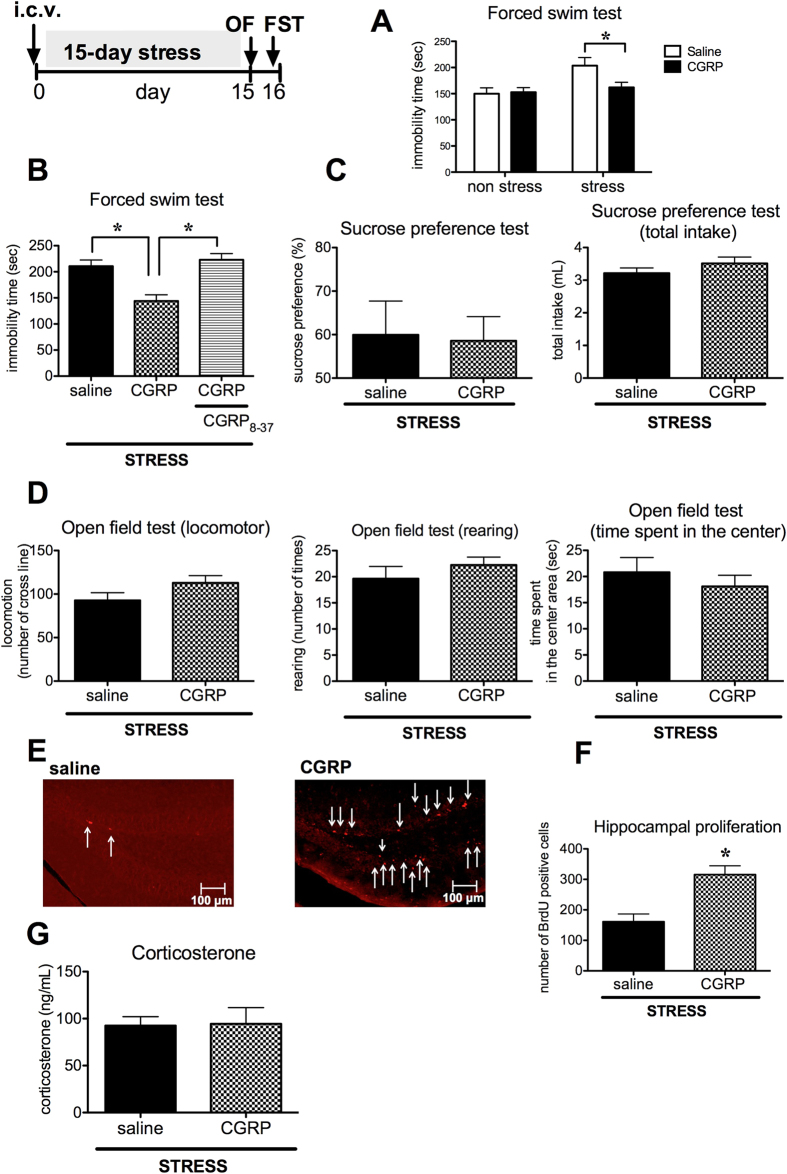
Exogenous CGRP administration rescues depression-like behavior. Mice were administered saline, CGRP (0.5 nmol), or CGRP in combination with the CGRP antagonist CGRP_8-37_ (0.5 nmol) i.c.v. before beginning the 15-day stress exposure. (**A**) Immobility times in the forced swim test (non-stress (saline n = 11, CGRP n = 10), stress (saline n = 9, CGRP n = 11)). (**B**) Immobility times in the forced swim test (saline n = 8, CGRP n = 8, CGRP + CGRP_8-37_ n = 8). (**C**) Sucrose preferences and total fluid intake in the sucrose preference test (saline n = 5, CGRP n = 5). No treatment significantly affected general behavior, locomotor activity, rearing activity, or time spent in the center area in the open-field test (**D**) (saline n = 8, CGRP n = 8). (**E**) Photomicrographs of the dentate gyrus showing representative BrdU-positive cells. Scale bar indicates 100 μm. Arrows show immunopositive cells. (**F**) The number of BrdU-positive cells (saline n = 4, CGRP n = 4). (**G**) Serum corticosterone level (saline n = 5, CGRP n = 5). Each bar indicates the mean ± S.E.M. *P < 0.05 vs. saline.

Next, we examined whether exogenous CGRP administration alters depression-like behaviors in stressed mice, using i.c.v. CGRP administration. First, mice were administered CGRP i.c.v. on day 15 after the final 3 h of stress exposure, followed by assessment of their performance in the forced swim test 24 h following CGRP treatment. CGRP treatment did not affect depression-like behavior (P = 0.1816, Fig. 3A). Next, after 15 days’ stress exposure, CGRP was administrated into the brain on day 16, and then behavioral tests were performed on day 30. There were no significant changes in immobility times in the forced swim test (P = 0.2102, Fig. 3B). In contrast, when a single dose of CGRP was administered on day 1, before beginning the 15-day stress exposure, CGRP significantly improved stress-induced increases in immobility times (F(1, 37) = 7.6, P = 0.009, Fig. 4A). Furthermore, co-treatment with the CGRP antagonist, CGRP_8-37_, abrogated this effect (F(2, 21) = 12.85, P < 0.01, Fig. 4B). However, in the sucrose preference test, CGRP administration did not significantly affect sucrose consumption and total fluid intake (P = 0.444, P = 0.1318, Fig. 4C). In the open field test, CGRP treatment did not significantly affect overall locomotor activity, rearing activity, or time spent in the center area (P = 0.06, P = 0.1823, P = 0.2286, respectively; Fig. 4D). Interestingly, the number of BrdU-positive cells in the granule cell layer of the dentate gyrus was significantly higher following CGRP treatment compared with saline treatment (P = 0.0035, Fig. 4E,F). The serum corticosterone level did not change (P = 0.4625, Fig. 4G). These data suggest that CGRP infusion induces antidepressant-like behaviors in the forced swim test as well as increasing hippocampal proliferation in stressed mice.

**Figure 5:**
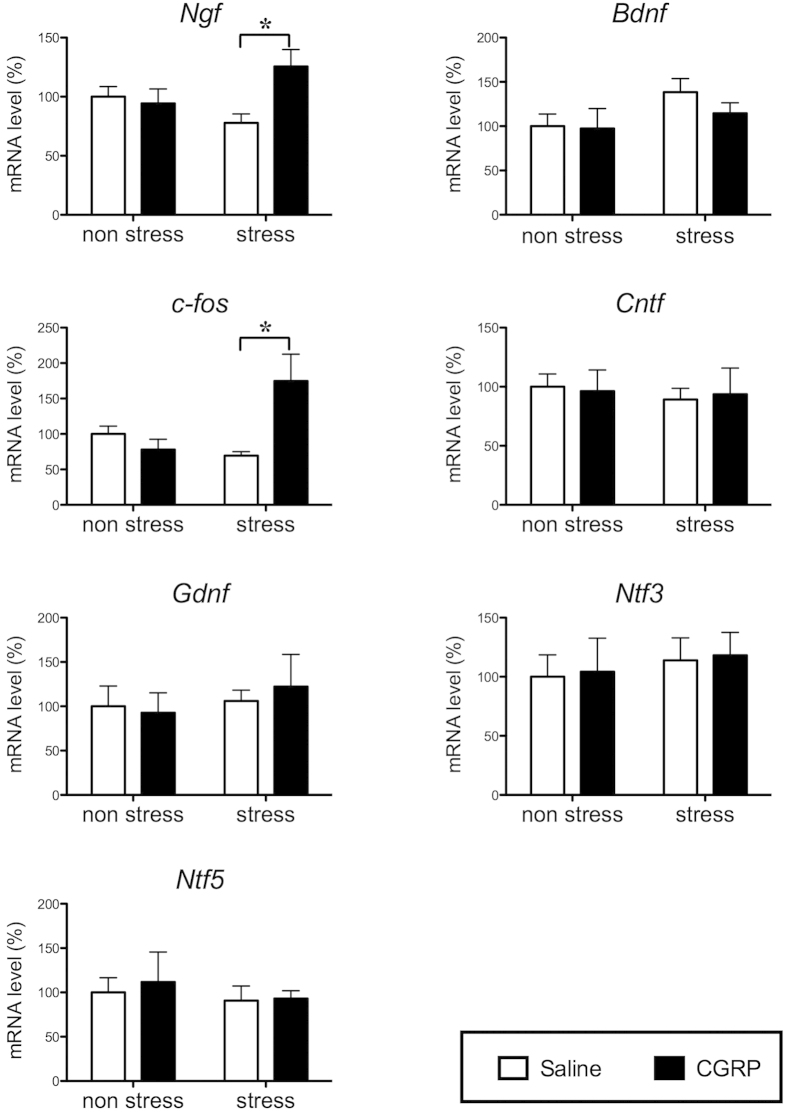
Effects of CGRP administration on mRNA level of neurotrophic factors or *c-fos* in the mouse hippocampus. Mice were administered saline or CGRP (0.5 nmol) i.c.v. before beginning the 15-day stress exposure. Quantitative real-time RT-PCR analysis showing the expression levels of *Ngf, Bdnf, c-fos, Cntf, Gdnf, Ntf3* and *Nt*f5 (non-stress (saline n = 9, CGRP n = 5), stress (saline n = 10, CGRP n = 9)). Each bar indicates the mean ± S.E.M. *P < 0.05 vs. saline.

**Figure 6:**
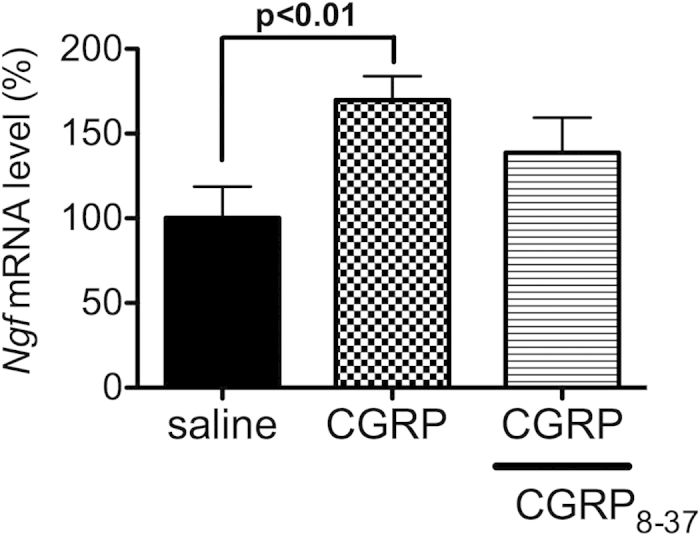
*Ngf* mRNA levels in mouse embryonic E14 hippocampal cell line. CGRP (100 nM) alone or CGRP with a CGRP antagonist, CGRP_8-37_ (100 nM) incubated for 60 min. CGRP_8-37_ was added 30 min before the addition of CGRP (saline n = 4, CGRP n = 4, CGRP + CGRP_8-37_ n = 4). Each bar indicates the mean ± S.E.M.

To define the mechanisms underlying the effects of CGRP in the mouse hippocampus, we focused on the expression of various neurotrophic factors, which are known to affect neuronal survival and plasticity of dopaminergic, cholinergic, and serotonergic neurons in the central nervous system^18^. We first determined whether the expression level of neurotrophic factors was changed by the administration of CGRP i.c.v. before beginning stress exposure (day 1 treatment). As shown in Fig. 5, CGRP pretreatment induced significant increases in *Ngf* (F(1, 29) = 5.59, P = 0.0250) and c*-fos* (F(1, 29) = 7.47, P = 0.0106) mRNA expression, but not *Bdnf*, *Cntf*, *Gdnf*, *Ntf3* or *Ntf5* mRNA expression. To examine whether *Ngf* expression was sensitive to exogenous CGRP administration, we assessed changes in *Ngf* mRNA expression levels in the mouse E14 hippocampal cell line. CGRP (100 nM) treatment significantly increased *Ngf* expression (F(2, 9) = 3.783, P < 0.01, Fig. 6). However, this change was not observed after co-treatment with the CGRF antagonist, CGRP_8-37_ (100 nM).

### The NGF receptor inhibitor K252a suppresses CGRP-induced antidepressant-like effects

**Figure 7:**
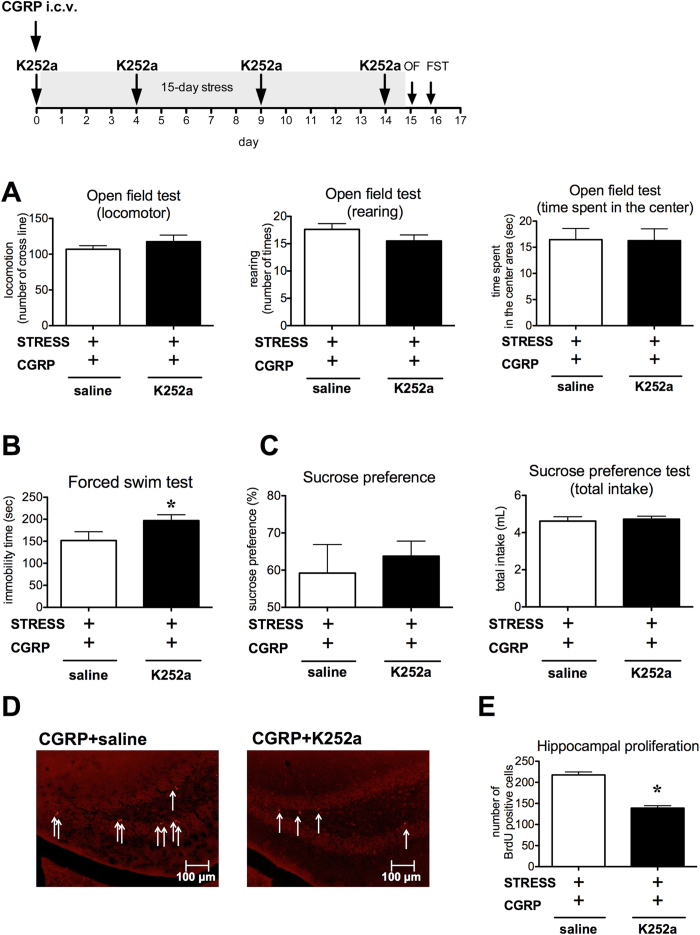
The NGF receptor inhibitor K252a suppresses CGRP-induced antidepressant-like effects. (**A**) No treatment significantly affected general behavior, locomotor activity, rearing activity, or time spent in the center area in the open field test (saline n = 8, K252a n = 8). (**B**) Immobility times in the forced swim test (saline n = 8, K252a n = 8). (**C**) Sucrose preference and total fluid intake in the sucrose preference test (saline n = 8, K252a n = 8). (**D**) Photomicrographs of the dentate gyrus showing representative BrdU-positive cells. Scale bar indicates 100 μm. Arrows show immunopositive cells. (**E**) The number of BrdU-positive cells (saline n = 4, K252a n = 4). K252a (25 μg/kg) was administered i.p. four times during stress exposure (day 1, 5, 10, 15). CGRP (0.5 nmol) was administered i.c.v. before beginning the 15-day stress exposure. Each bar indicates the mean ± S.E.M. *P < 0.05 vs. saline.

To determine whether CGRP–mediated NGF was responsible for improvements in depression–like behavior, we examined the effects of K252a treatment. K252a is well known as a broad–spectrum inhibitor of tyrosine kinase activity through Trk receptors^20^. The K252a treatment did not significantly affect general motor behavior in the open field test (locomotor activity (P = 0.1641), rearing activity (P = 0.0923) and time spent in the center area (P = 0.4756)) when compared with saline treatment (Fig. 7A). The CGRP-dependent antidepressant-like effect was significantly inhibited by K252a treatment in the forced swim test (P = 0.0407, Fig. 7B). In contrast, sucrose preferences and total fluid intake were not significantly affected by combined CGRP and K252a treatment (P = 0.2968, P = 0.3581, Fig. 7C). The CGRP-induced increased hippocampal cell proliferation in stressed mice was blocked in K252a-treated animals (P = 0.0001, Fig. 7D,E).

## Discussion

Changes in neurotrophic factors are involved in the pathogenesis of depression. However, the relationship between CGRP, a neuropeptide, and depression-like behavior remains unclear. In this study, we used chronically stressed mice to investigate whether CGRP can affect behavior. We show that pre-administration with only one treatment of CGRP into the brain prior to stress exposure suppresses stress-induced depression-like behavior in mice. In contrast, CGRP administration after stress exposure does not change depression-like behavior. Our findings elucidate a novel function for CGRP, suggesting that CGRP pre-administration may protect against depression caused by a subsequent severe stressful situation.

To our best knowledge, this is the first report exploring CGRP’s ability to improve depression-like behavior in a stress model in mice. Our present findings are consistent with reports that in the maternal deprivation stress model in rats, CGRP-like immunoreactivity was decreased in the hippocampus and occipital cortex^21^. Furthermore, central administration of CGRP induced active behavior in the forced swim test in AKR and C57BL/6 mice^22^. These reports support our hypothesis that CGRP is involved in stress responses. However, some researchers have reported that CGRP-like immunoreactivity was increased in cerebrospinal fluid^10^ or plasma^23^ of depressed patients. Furthermore, administration of CGRP i.c.v. prolonged immobility time in the forced swim test in out-crossed mice^24^. These conflicting findings might be due to differences between CGRP expression in the brain and its release to peripheral sites, or because of mouse strain differences. Sink *et al.* have reported that CGRP i.c.v. evoked anxiety behavior^8^. In the present study, the time spent in the center area in the open field test, which evaluate anxiety-like behavior, did not change in stressed mice when compared to control. Based on our data, we conjecture 15-day stress mice did not engender anxiety-like behavior. Furthermore, Sink *et al.* examined anxiety behavior on the day of CGRP administration. In contrast, present study evaluated depression-like behavior 15 days after CGRP administration. In Fig. 4D showed pre-treatment CGRP did not show any significant different in the time spent in the center area in stressed mice. Thus, it is likely that CGRP antidepressant effect is not instantaneous but sequential effect.

The chronic stress paradigm used in this study was based on a restraint stress model. Previous studies have shown that 10 days’ immobilization in a polyethylene tube for 2 h produces depression–like behavior^25^ and changes neuronal morphology^26^. In contrast, another report has shown that this protocol only slightly affects behavior^27^. These discrepant results suggest that inducing stress solely by restraint may be insufficient to affect mouse behavior. Therefore, we combined restraint and water immersion stress. Using a 15-day mouse stress model, we provide evidence for depression-like behavior using the forced swim and sucrose preference tests, the measurement of serum corticosterone levels and proliferation in the hippocampus by BrdU immunostaining. In addition, significant decreases in the hippocampal mRNA levels of *c-fos,* as a marker of neuronal activation, *Cgrp* and *Ngf* were observed. In contrast, the *Bdnf* mRNA level was significantly increased in stressed mice. It has been reported that the level of hippocampal BDNF is correlated with depression^28^. Interestingly, after 7 days of stress exposure, mice exhibited increased immobility time in the forced swim test, decreased *Bdnf* mRNA expression levels and decreased hippocampal proliferation (data not shown). Further clarification of the role of BDNF over the time course of the 15-day stress model is required.

It is known that increased plasma corticosterone levels induce depression–like behavior^29^. In the present model, there is no correlation between CGRP–induced antidepressant effects and the levels of corticosterone (Fig. 4G). However, we found that *Ngf* mRNA expression was increased in the hippocampus of stressed mice treated with CGRP. This effect may be attributable to the direct actions of CGRP, because in the E14 mouse hippocampal cell line, CGRP administration induced increases in *Ngf* mRNA. Furthermore, K252a blocked the antidepressant-like effects of CGRP, indicating that CGRP-induced NGF may rescue depression-like behavior. In support of this hypothesis, NGF treatment recovered depression-like behavior with an increase in the level of monoamine neurotransmitters in the brain in rats subjected to mild chronic stress^15^. Furthermore, we demonstrated that hippocampal proliferation was increased by CGRP. Other neurotrophic factors (BDNF, CNTF, and GDNF) may be involved in CGRP-mediated behavioral improvements, because K252a also blocks the BDNF receptor (trkB). CGRP enhances release of BDNF in rat trigeminal ganglion neurons^30^. However, our current data indicate that CGRP treatment did not induce *Bdnf* mRNA expression in the E14 mouse hippocampal cell line and mouse hippocampus. This discrepancy could result from different processes in the hippocampus and trigeminal ganglion. We cannot yet explain why a single CGRP administration given before beginning the stress exposure maintained the level of *Ngf* mRNA increases for 15 days. In the present study, we demonstrated that exogenous CGRP post-stress exposure on day 15 did not affect depression-like behavior (Fig. 3A). However, exogenous CGRP pre-stress exposure on day 1 improved swimming time in the forced swim test and hippocampal proliferation (Fig. 4). Therefore, we hypothesize that the 2-week period plays an important role in CGRP-mediated behavioral changes. To test this hypothesis, we performed the 15-day stress exposure and on day 16, CGRP was administrated into the brain, then behavioral tests were performed on day 30. There were no significant changes in immobility time in the forced swim test, indicating that it is important to administer CGRP before the stress exposure. We could not rule out that CGRP facilitated learning and memory formation, because CGRP can prolong passive avoidance latencies in the rat^9^. Consistent with this report, our current results also showed increases in hippocampal cell proliferation and *Ngf* mRNA levels after pretreatment with CGRP. This hypothesis seems reasonable, because CGRP given i.c.v. did not influence anhedonic behavior. In the present study, we used water immersion to induce stress, which can affect immobility times in the forced swim test. However, if CGRP improves learning and memory, immobility times in the forced swim test would be expected to be longer. Our stress model involved water immersion with restraint for 3 h over 15 consecutive days, which is enough to engender learned helplessness and eliminate attempts to escape from the water. We speculate that CGRP pretreatment acts as ischemic preconditioning to protect brain cells. In the heart, ischemic preconditioning, which involves repeated short episodes of ischemia, protects the myocardium by releasing bradykinin or adenosine from ischemic cells^31^. It has been widely reported that CGRP plays a protective role in preconditioning isolated rat hearts and mice brain^32^,^33^. In addition, endogenous CGRP mediates the protective effect of cell damage^34^. These data support our hypothesis that CGRP pre-administration into the brain induces NGF expression that protects against stress-induced depression-like behavior.

We found that CGRP prevented the behavioral effects induced by 15–day stress exposure in the forced–swim test. However, other types of depression–like behavior, including sucrose preferences, were not affected by CGRP administration. This selective effect of depression–like behavior may have resulted from the specific functions of CGRP. Our data suggest that hippocampal cell proliferation and the levels of NGF are involved in the behavioral performance in the forced swim test, whereas anhedonic behaviors may be caused by dysregulation of other factors in the hippocampus. In fact, the activity of the hippocampal network (the dentate gyrus to CA1) is linked to depression–related behavior in the forced swim test^35^. In addition, other reports suggest that decreased sucrose consumption, as indicative of an anhedonic state, is associated with decreases in serotonin transporter expression in mouse hippocampus^36^. Moreover, Aβ40, BACE1, and Т phosphorylation—which are Alzheimer’s disease-related markers—increase in anhedonic rat hippocampus^37^.

In conclusion, the present findings indicate that pre-administration of CGRP influences depression-like behavior in mice through modification of NGF expression in the hippocampus. Our findings provide a novel function for CGRP, and a potential target for novel treatments for depression.

## Methods

### Animals

All animal procedures were carried out in accordance with the Guide for the Care and Use of Laboratory Animals as published by the Japanese Association for Laboratory Animal Science. All experiments were approved by the Animal Care and Use Committee of the Okayama University of Science. According to these guidelines, efforts were made to minimize the number of animals used and their suffering. Six-week-old C57BL/6 male mice were purchased from Shimizu Experimental Animals (Shizuoka, Japan) and habituated to the colony for 2 weeks. All animals were housed in the Animal Research Center of Okayama University of Science at a controlled ambient temperature of 22 °C with 50 ± 10% relative humidity and a 12 h light/dark cycle (lights on at 7:00 AM). Animals were group-housed. Each home cage contained five to six mice.

### 15-day stress procedure

**Figure 8:**
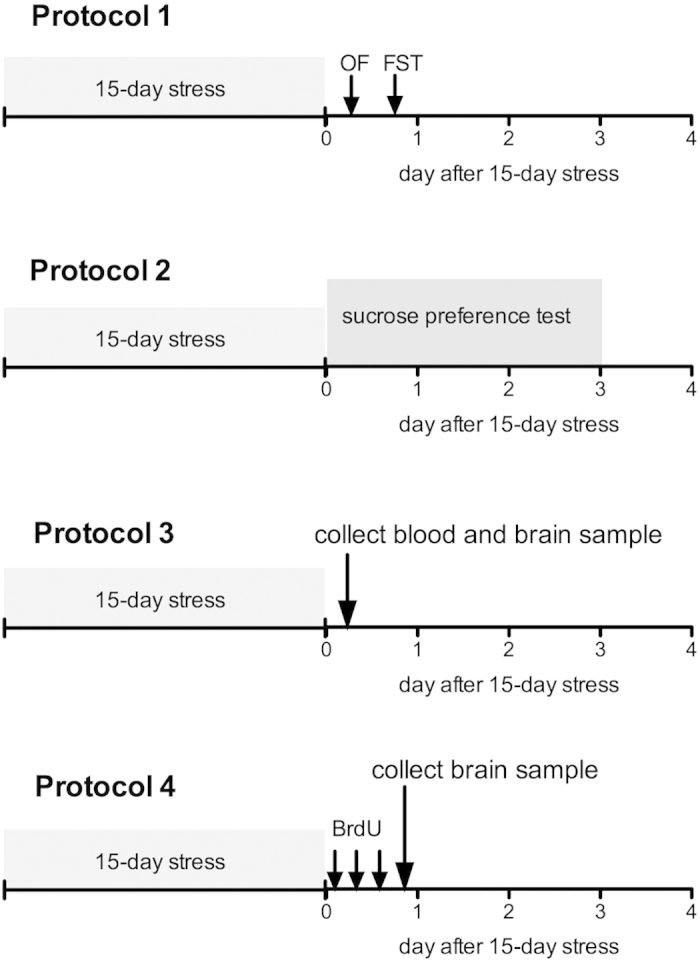
Experimental timelines. Protocol 1: to observe spontaneous behavior and depression-related behavior, open field test (OF) and forced swim test (FST) performances were assessed on the day following the 15-day stress exposure (day 16). Protocol 2: to observe depression-related behavior, the sucrose preference test was assessed days 1–3 after the 15-day stress exposure (day 16–18). Protocol 3: to observe serum corticosterone and mRNA levels of neurotrophic factors, blood and brain samples were collected at the end of the 15-day stress exposure from mice that had not undergone behavioral testing. Protocol 4: to study cell proliferation in the hippocampus, a single injection of 5-bromo-2-deoxyuridine (BrdU; 50 mg/kg, i.p.), was administered 3 times on day 15 of the 15-day stress exposure.

Animals (8 weeks old) in the stress groups were subjected to 3 h restraint for 15 days starting at 10:00 AM daily, using a protocol modified from a previously reported chronic stress model^38^. Each mouse was placed in a modified 50 mL polyethylene tube fitted with multiple air holes and immersed in a constant temperature water bath (28 °C) using a tube stand. In the morning and afternoon of day 16, after 15 days’ exposure to stress, we carried out open field and forced swim tests. The sucrose preference test was administered for 3 days following the 15-day stress exposure. To determine whether the 15-day stress exposure resulted in changes in serum corticosterone levels and the mRNA expression level of neurotrophic factors, blood and brain samples were collected in subjects that did not undergo behavioral testing. Furthermore, to evaluate hippocampal proliferation, brain samples were also collected from mice that did not undergo behavioral testing (Fig. 8).

### 5-Bromo-2-deoxyuridine (BrdU) labeling

To observe cellular proliferation in the hippocampus, *in vivo* BrdU (50 mg/kg, Sigma, Tokyo, Japan) labeling was performed. On day 16, the animals were deeply anesthetized with sodium pentobarbital (50 mg/kg, i.p.) and transcardially perfused with 0.9% (w/v) saline, followed by 4% (w/v) paraformaldehyde and 0.35% (v/v) glutaraldehyde in 0.1 M sodium phosphate buffer (PB), pH = 7.4. The brains were post-fixed overnight in 4% (w/v) paraformaldehyde in 0.1 M PB and cryoprotected in 15% (w/v) sucrose in 0.1 M PB with 0.1% (w/v) sodium azide overnight at 4 °C. The brains were sectioned (20 μm) using a cryostat, and immunocytochemistry was performed on free-floating sections. Sections were incubated in a monoclonal rat anti-BrdU antibody (1:300; Abcam plc, Tokyo, Japan) overnight at 4 °C. After washing, sections were incubated for 60 min at room temperature with Alexa Fluor 594 goat anti-rat IgG (1:300, Invitrogen™, Tokyo, Japan). Following several washes, sections were cover-slipped and observed under a fluorescent microscope (EVOS FLoid Cell Imaging Station, Life Technologies, Tokyo, Japan). All morphological analyses were performed on blind coded slides. To evaluate cell proliferation, BrdU-positive cells in every 12th bilateral section (240 μm interval) throughout the subgranular zone cell layer were counted. The number of BrdU positive cells was multiplied by a factor of 12 to estimate the total number of BrdU positive cells.

### Analysis of serum corticosterone

Blood samples were collected between 3:00 PM and 4:00 PM, from the caudal vein under light anesthesia immediately before brain fixation. Serum corticosterone was measured using a competitive ELISA kit (Assaypro LLC. MO. USA).

### Behavioral assessments

#### Open field test

Mice were placed in the center of a circular open field chamber (57.5 cm diameter, 32 cm high). The floor was divided into 19 sections, each section with almost the same area. The center area was defined by a circle with radius 35.5 cm (3957 cm^2^). Locomotor activity was scored as a line crossing when a mouse removed all four paws from one section and entered another. All animals’ behavior was videotaped by digital camera. Line crossings, rearing activity and the time spent in the center area were measured over the course of 3 min using a stopwatch and counter.

#### Forced swim test

Animals were placed in a 1 L Griffin beaker (15 cm high × 11 cm in diameter filled with 28 °C water to a depth of 10 cm) for 6 min, and the duration of floating (i.e., the time during which the animal made only the small movements necessary to keep its head above water) and the latency to the time when the animal became immobile were scored. Immobility time was analyzed during the last 5 min of the test.

#### Sucrose preference test

The sucrose preference test was based on the methods described by Jiang *et al.*^39^. Animals were habituated to drinking water from two bottles for 3 days before the last experimental day. Two pre-weighed bottles, one containing tap water and the other containing a 1% (w/v) sucrose solution, were presented to each animal for 1 h. Then, the animals underwent the second sucrose preference test for 1 h following 6 h rest. In this test, the position of the water and sucrose bottles (left or right) was switched. The sum of the water plus sucrose intake was defined as the total intake, and a sucrose preference was expressed as the percentage of sucrose intake relative to the total intake.

### RNA extraction

The animals were sacrificed on day 16, 24 h after the last stress treatment, by administration of an overdose of pentobarbital-Na (100 mg/kg). Total RNA was extracted from the hippocampus and placed in RNAlater (Life Technologies Co., Tokyo, Japan), and stored at −30 °C. Total RNA was extracted using the RNeasy Plus Micro kit (Qiagen, Tokyo, Japan). After extraction, RNA samples were dissolved in nuclease-free water (Qiagen) and the optical density values of each sample were determined using an absorption meter (Shimadzu Co., Tokyo, Japan). We performed reverse transcription using M-MLV reverse transcriptase (Wako Pure Chemical Industries, Ltd., Osaka, Japan) according to the manufacturer’s protocol. Specificity of amplification was verified by the monophasic character of the melting curve generated for each amplification product by the Eco Real-Time PCR System (Illumina Inc., Tokyo, Japan) at the end of the PCR.

### Quantitative analysis by real-time PCR

The reverse-transcribed mixture was used as a template for subsequent real-time PCR. Real-time PCR was performed using Power SYBR Green PCR Master Mix (Life Technologies) and analyzed with the Eco Real-Time PCR System (Illumina Inc.). Primers, designed by the authors, were based on the coding sequences of mouse genes deposited in GenBank. The data were analyzed using the mean threshold cycle equation. The primer information is shown in Table 1. Glyceraldehyde-3-phosphate dehydrogenase (*Gapdh*) served as an internal control. The threshold cycle values for both the target (*Cgrp, Bdnf, Cntf, Gdnf, Ngf, Ntf3, Ntf5, c-fos*) and internal control (*Gapdh*) genes were determined. The fold change of each gene was normalized to *Gapdh* and was calculated for each sample, relative to the expression in the control samples.

### Drug treatments

#### Intracerebroventricular (i. c. v.) administration

Rat CGRP (Sigma; 0.5 nmol) and CGRP_8-37_ (Sigma; 0.5 nmol) were diluted in saline. Inhaled diethyl ether was used for brief anesthesia during i.c.v. injections. Drug administration was by direct injection into the right lateral ventricle through the intact scalp aiming at 1 mm posterior to the bregma and 1 mm right from the midline as described previously^40^.

In Fig. 3A, the effect of CGRP i.c.v. on depression-like behavior in the forced swim test was assessed 24 h after saline or CGRP i.c.v. following the 15-day stress exposure. In Fig. 3B, saline or CGRP was administrated on day 16 following the 15-day stress exposure and behavioral testing. The forced swim test was assessed on day 30. In Fig. 4, saline, CGRP or CGRP with CGRP_8-37_ (CGRP antagonist) was administrated on day 1 (3 h before stress exposure), and then behavioral testing was assessed 24 h after the 15-day stress exposure.

In Fig. 7, K252a (25 μg/kg, Sigma), a broad-spectrum tyrosine kinase antagonist, was used. To avoid K252a excessive action, we used low concentration of K252a^39^ and administrated itraperitoneally four times during the stress exposure (days 1, 5, 10 and 15).

### Cell culture

Mouse embryonic E14 hippocampal cells (CELLutions Biosystems Inc., Ontario, Canada) were cultured in Dulbecco’s Modified Eagle Medium (DMEM) (Gibco Invitrogen, Tokyo, Japan) containing 10% fetal bovine serum (FBS) (Gibco Invitrogen), 100 U/mL penicillin and 100 μg/mL streptomycin (Gibco Invitrogen). Cells were maintained at 60–70% confluency at 37 °C in a humidified incubator with 5% CO_2_ and air.

To examine expression of *Ngf* , cells were synchronized in 1% FBS-DMEM overnight at 60% confluency. The cells were treated for 60 min w16ith 100 nM CGRP alone or CGRP with CGRP antagonist, CGRP_8-37_ (100 nM). CGRP_8-37_ was added 30 min before the addition of CGRP. The cells were dissolved in denaturing solution (4 M guanidinium thiocyanate, 25 mM sodium citrate, 0.5% N-lauroylsarcosine and 0.1 M beta-mercaptoethanol) and gene expression levels analyzed by real-time PCR.

### Statistical analysis

All data are expressed as the mean ± S.E.M. Comparisons between two values were analyzed using the Student’s t-test. Analysis of variance followed by Dunnett’s multiple comparison test was used to determine statistical significance where appropriate. Otherwise, differences were analyzed by two-way analysis of variance followed by Bonferroni posttests. A P value < 0.05 was considered statistically significant.

## Additional Information

**How to cite this article**: Hashikawa-Hobara, N. *et al.* Calcitonin gene-related peptide pre-administration acts as a novel antidepressant in stressed mice. *Sci. Rep.* **5**, 12559; doi: 10.1038/srep12559 (2015).

## Figures and Tables

**Table 1:** Oligonucleotide sequences for real-time PCR amplification.

	Forward	Reverse
*Cgrp*	CCTGCAACACTGCCACCTGCG	GAAGGCTTCAGAGCCCACATTG
*Ngf*	CACAGCCACAGACATCAAGGGC	CCTGCTTCTCATCTGTTGTC
*Gdnf*	CCAGAGAATTCCAGAGGGAAAGGTCGC	GATCAGTTCCTCCTTGGTTTCATAGC
*Cntf*	ACAGTGGACTGTGAGGTCTATCC	GGAGACAGAGGCAAGAGTTAAGAG
*Ntf3*	GGTAGCCAATAGAACCTCACCAC	GTCACACACTGAGTACTCTCCTC
*Ntf5*	CACTCCTGTTCTCTCCTCCTTTTC	GGAGACAAGAGGTCCCACTCAGG
*Bdnf*	TGGCTGACACTTTTGAGCACGTC	GCTCCAAAGGCACTTGACTGCTGA
*c-fos*	GGTGAAGACCGTGTCAGGAGGCAG	GCCATCTTATTCCGTTCCCTTCGG
*Gapdh*	GCCATTTGCAGTGGCAAAGTGG	GATGGGCTTCCCGTTGATGACAAGC
